# Consensus statement on physical rehabilitation in children and adolescents with osteogenesis imperfecta

**DOI:** 10.1186/s13023-018-0905-4

**Published:** 2018-09-10

**Authors:** Brigitte Mueller, Raoul Engelbert, Frances Baratta-Ziska, Bart Bartels, Nicole Blanc, Evelise Brizola, Paolo Fraschini, Claire Hill, Caroline Marr, Lisa Mills, Kathleen Montpetit, Verity Pacey, Miguel Rodriguez Molina, Marleen Schuuring, Chantal Verhille, Olga de Vries, Eric Hiu Kwong Yeung, Oliver Semler

**Affiliations:** 1 Unireha, University of Cologne, Center of Prevention and Rehabilitation, Cologne, Germany; 2 University of Cologne, Children’s Hospital, Kerpenerstraße 62, 50931 Cologne, Germany; 3 ACHIEVE, Center for Applied Research, Faculty of Health, University of Applied Sciences Amsterdam, Amsterdam, The Netherlands; 4 Department of Rehabilitation, Academic Medical Centre, University of Amsterdam, Amsterdam Movement Sciences, Amsterdam, The Netherlands; 5 Hospital for Special Surgery, NYC, NY, USA; 6 Children’s Hospital, Toulouse, France; 7 Medical Genetics Service, Hospital de Clinicas de Porto Alegre, Porto Alegre, Brazil; 8 National Resource center for rare disorders. Part of the National Advisory Unit on Rare Disorders (NKSD), Sunnaas Rehabilitation Hospital, Nesodden, Norway; 9 IRCCS Eugenio Medea, Bosisio Parini (LC), Italy; 10 Sheffield Children’s NHS Foundation Trust, Sheffield, UK; 11 Bristol Royal Hospital for Children, University Hospitals Bristol NHS Foundation Trust, Bristol, UK; 12 Shriners Hospital for Children, Montreal, Quebec, Canada; 13 The Children’s Hospital at Westmead, Sydney, Australia; 14 Macquarie University, Sydney, Australia; 15 AHUCE and Fundacion AHUCE, Madrid, Spain; 16 Child development and exercise center, Wilhelmina´s Children Hospital, University Medical Center Utrecht, Utrecht, The Netherlands; 17 University of Leuven, Leuven, Belgium; 18 The University of Hong Kong, Hong Kong, China

**Keywords:** Osteogenesis imperfecta, Physiotherapy, Occupational therapy, Mobility, Rehabilitation

## Abstract

On the occasion of the 13th International Conference on Osteogenesis imperfecta in August 2017 an expert panel was convened to develop an international consensus paper regarding physical rehabilitation in children and adolescents with Osteogenesis imperfecta. The experts were chosen based on their clinical experience with children with osteogenesis imperfecta and were identified by sending out questionnaires to specialized centers and patient organizations in 26 different countries. The final expert-group included 16 representatives (12 physiotherapists, two occupational therapists and two medical doctors) from 14 countries. Within the framework of a collation of personal experiences and the results of a literature search, the participating physiotherapists, occupational therapists and medical doctors formulated 17 expert-statements on physical rehabilitation in patients aged 0–18 years with osteogenesis imperfecta.

## Aim of the position paper

The purpose of this consensus paper is to collect expert knowledge as well as the evidence in the literature regarding physical rehabilitation in children and adolescents with Osteogenesis imperfecta (OI) in order to develop evidence based recommendations. Due to the difficulties of performing high level clinical trials in the field of rehabilitation and among patients with rare diseases, these consensus statements are intended as guidelines for the individual therapist and should be adapted to the needs of each patient. Particular attention must be paid to the severity of the disease in the individual child, as this will influence the therapeutic options and goals. We focused on children from infancy to adolescence (0–18 years of age) because this is the age when therapy is most critical for promoting the development of motor skills and independence in later life. We searched for all strategies currently used to improve motor function in these children with OI, including physiotherapy, occupational therapy and other types of rehabilitative approaches. For this paper we used the classification system introduced by Sillence (OI types I, III, IV, etc.) because it is based on clinical findings relevant for rehabilitation. We also used the terms; mild, moderate or severely affected as these descriptions correspond to the initial Sillence types and are clinically more meaningful than classifications based on pathophysiology or genetics [1].

We excluded any literature on medical, surgical or psychological treatments despite their importance to children with OI [2]. Needless to say surgery and medical treatment with bisphosphonate do influence the outcomes of therapeutic programs; however these treatments are not the focus of this paper and recommendations about these topics can be found in the literature [1].

We narrowed the recommendations to rehabilitation approaches directed at improving muscle performance, mobility and self-care which may then lead to better functional activity and participation. We attempted to include all approaches described in the literature, but this does not prohibit the individual therapist from investigating other approaches for a specific individual. Any treatment approaches should be guided by evidence based practice, clinical assessment and validated and reliable assessment tools followed by clinical reasoning and decision making.

## Introduction

OI is the most common hereditary form of bone fragility in childhood with an estimated incidence of 1:20.000 births [2]. The phenotype varies substantially ranging from those showing minimal symptoms (one to two fractures per year) until puberty to those who die during the first few days or weeks of life due to rib fractures and lung hypoplasia [3]. Most cases are due to an impaired production of collagen caused by mutations in *COL1A1/A2*. Milder types are frequently due to stop mutations resulting in a quantitative defect of collagen, while other mutations can produce impaired collagen [1]. There is no clear genotype – phenotype correlation which could help in counselling or treatment of the individual child [4].

Recurrent fractures during childhood from minimal trauma are the most prominent sign of OI. Deformities of the extremities, spine and varying degrees of short stature can appear in moderate to severe forms of the disease. In addition, any affected child may also present with extra-skeletal signs such as muscle weakness, joint hypermobility, involvement of teeth (dentinogenesis imperfecta) and hearing loss.

Currently the management is based on medical treatment with anti-resorptive drugs to reduce bone resorption by osteoclasts, in conjunction with surgical treatment to stabilize fractures and to correct deformities [5]. This can be done either with immobilization for fractures or surgery to insert intramedullary rods in the case of deformities [6]. In addition to these treatments, training of muscle function, mobility and self-care is the most important aspect to improve independence and quality of life (QoL) in patients with OI [7, 8].

This paper will provide the most up to date rehabilitation management of children and adolescents with OI. The consensus statements are intended to assist physiotherapists, occupational therapists and other health care professionals to establish treatment goals and develop a treatment plan for individuals with OI.

## Methods

The first step in this process was a review of relevant medical and therapeutic literature from January 1970 to April 2017 using Pubmed, Pedro and hand-searching.

Based on these findings, a questionnaire was created with the primary goal of obtaining an overview of the experience and the therapeutic approaches used worldwide in the rehabilitation of children with OI.

The questionnaire was sent to experienced physiotherapists, occupational therapists and physiatrists around the world. These therapists were identified through the literature review, patient support organizations (eg.Osteogenesis imperfecta Federation Europe (OIFE)) and by screening the proceedings of previous international conferences. Therapists were invited to share the questionnaire with colleagues also working with the OI population.

Ninety-nine questionnaires were sent to therapists in 26 different countries. Fifty-three questionnaires were returned from 17 countries [North America (7), South America (4), Europe (38), Africa (0), Asia (2), Australia (2)]. Fifty responses were from physiotherapists and three from occupational therapists.

From the responses the most experienced experts from each country were identified, based on years of experience, number of children treated and amount of research activity. The one to two most experienced respondents from each country were asked to form an expert international group. It was aimed for a maximum from 2 people from one country with the same occupation. In countries with more than one qualified therapist those were asked to agree to one representative of their country in the group.

The final expert group included 16 representatives; 12 physiotherapists, two occupational therapists and two medical doctors from 14 countries. The two doctors are RE how is a rehabilitation specialist and active in the area of physical therapy in OI since decades and OS working at a pediatric rehabilitation center for children with OI and chairing this consensus group. The therapists all worked in highly specialized hospital (most attached to a university) and the therapists have all seen more than 30 OI children during their career and have at least more than 5 years of experience in OI.

Based on the literature review and the results of the questionnaire, six topics of interest (musculoskeletal, spine, infancy &development, mobility, self care & upper extremity, therapy following surgery) were identified by the expert group during the first telephone conference. The different parts were chosen based on anatomical structures (spine) or in respect to the content. Therefore topics like therapy of upper extremities and self-care were combined as well as lower extremities and mobility. The therapy of infants focus on the improvement of development and where fused. Treatment after surgery had some special aspects and was therefore kept separately. In a telephone conference the experts were assigned to working groups on these topics based on their experience. The working groups were provided with the results of the questionnaires and with the literature on their topic. Each group summarized the pertinent publications, explored the current standard of care and shared their expert experience. These summaries were discussed by the entire expert panel and one to three consensus statements for each topic were proposed. The final draft was discussed by all experts during the Consensus-Meeting held during the 13th International Conference on Osteogenesis imperfecta (Oslo, August 2017). The statements were revised until consensus was reached.

## Summary of clinical trials

**Fig. 1:**
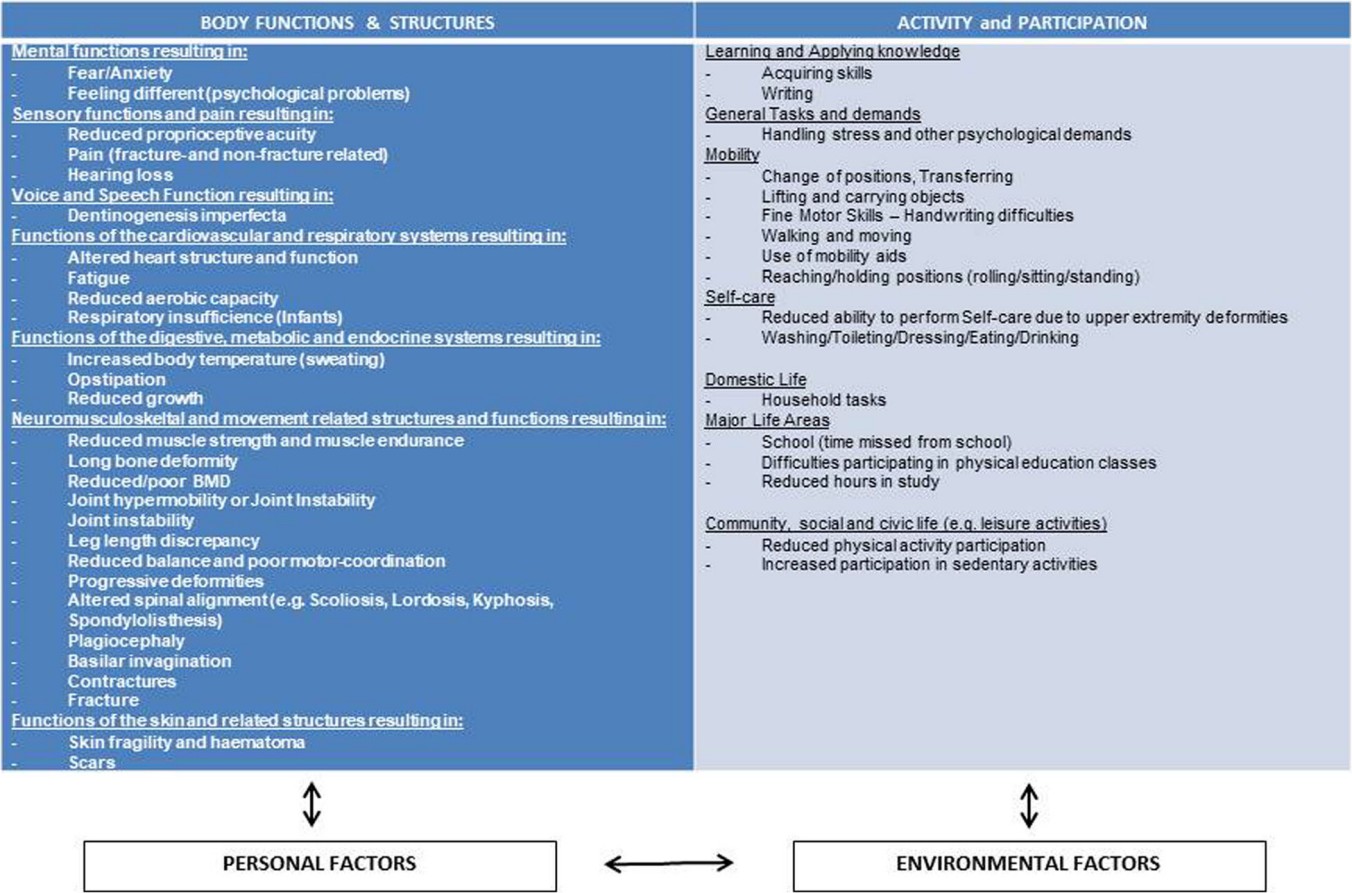
Results of literature search

The literature search revealed only 4 papers describing randomized or longitudinal studies or providing data of a sufficient number of patients. These papers are summarized below. The results of the search are shown in Fig. 1. It must be acknowledged that double blind trials are rarely possible when studying therapeutic interventions [9]*.*

Gerber et al. investigated the effects of withdrawal of hip-knee-ankle-foot orthoses (HKAFO) on activity and ambulation in a prospective, randomized cross-over matched pair trial in ten moderate to severely affected children over a period of 32 months (16 months with braces and 16 months without braces). The results showed that removing braces had no effect on muscle strength or independence but the fracture rate increased. Children and families reported feeling safer when using the braces. This might explain the positive effect of bracing in this small trial [10]. However this study was performed prior to the use of bisphosphonates in severely affected patients. Therefore these results might not be comparable for children nowadays who are now treated form an early age with bisphosphonates.

In 2004 Engelbert et al. investigated functional abilities in a prospective study with 4 years of follow-up in 49 children not receiving a specific intervention. A decrease of range of motion (ROM) of the joints of the lower extremities occurred in all patients regardless of the severity of the disease. Type of OI and total muscle strength were the only significant predictors for both level of ambulation and the dependence on support for daily activities. Additionally he described that body weight was significantly lower in the group with better ambulation, whereas children with a reduced level of ambulation had significantly higher body weight. It is not possible to ascertain whether the increased body weight was the reason for the reduced ambulation or if the immobility caused the increased weight [7].

Van Brussel et al. investigated the effects of a physical training program on exercise capacity and muscle force. Thirty-four ambulatory children with OI type I or IV were prospectively assigned to either a 12-week exercise program or usual care. The intervention consisted of 6 weeks of twice weekly exercise sessions followed by weekly home-based exercises. At the end of the trial the intervention group showed significantly improved muscle force and peak oxygen consumption however the gains faded were not maintained following the cessation of once training ended [11].

The effect of a rehabilitation approach which included whole body vibration and several other treatment strategies was investigated in a retrospective review by Hoyer-Kuhn et al. This program consisted of a 3-week inpatient stay and a whole body vibration training at home over a period of 6 months. Data from 53 children with different severities were analysed and demonstrated that an intensive training had a positive effect on mobility even after vibration training ended. Positive effects were shown using the Gross Motor Function Measure and 1- and 6-min-walking tests. No effect was seen on fracture rate or bone mass acquisition [12].

## General aspects

**Fig. 2:**
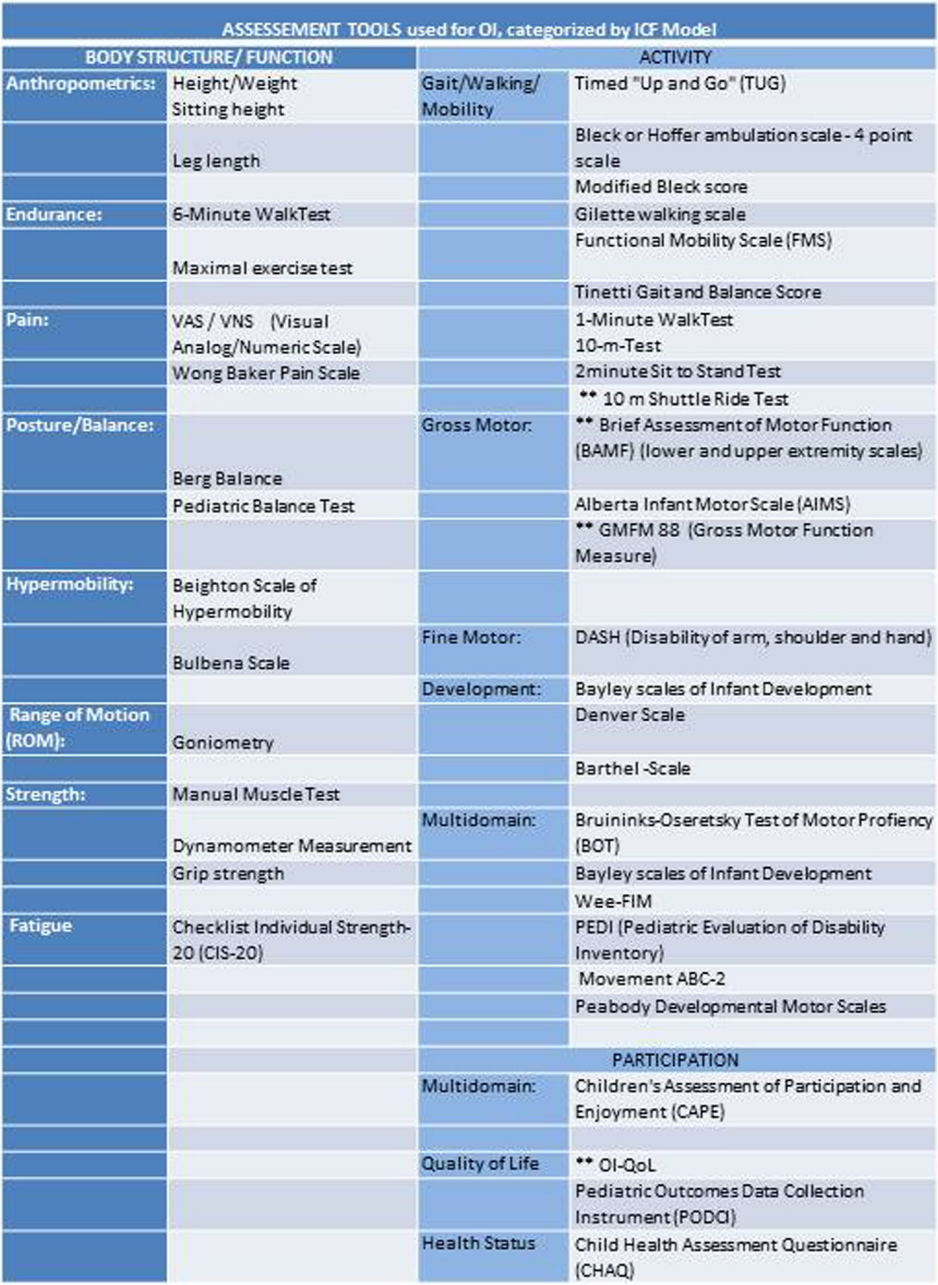
Proposed ICF based concept of rehabilitation adapted to children and adolescents with OI

Due to the complexity of the symptoms in OI, the International Classification of Functioning, Disability and Health (ICF) was adopted as an overall framework for this project [13]. Disability, according to the World Health Organization, is an umbrella term covering functions, activities and participation, as well as environmental and personal factors (WHO, 2015). In children, adolescents and adults, impairments in the ICF domain of body and function have an impact not only on the structural aspects (e.g. skeletal deformities, weak muscles, fractures) but may also result in decreased functional capacity and restrictions in participation. Depending on the severity of OI, young people may have difficulties with activities of daily living, sport and leisure activities as well as participation in society. A diagram of the ICF adapted for OI is illustrated in Fig. 2.

Interdisciplinary treatment strategies must be designed based on clinical findings with knowledge of the interactions between the ICF domains. The treatment should “*include all aspects of expert practice, including knowledge, core values, clear clinical reasoning and excellent clinical practice skill focused on providing high-quality, patient-centered care”* [13]*.* Based on the needs of the children and caregivers, the intervention should be focused on these topics.

**Fig. 3:**
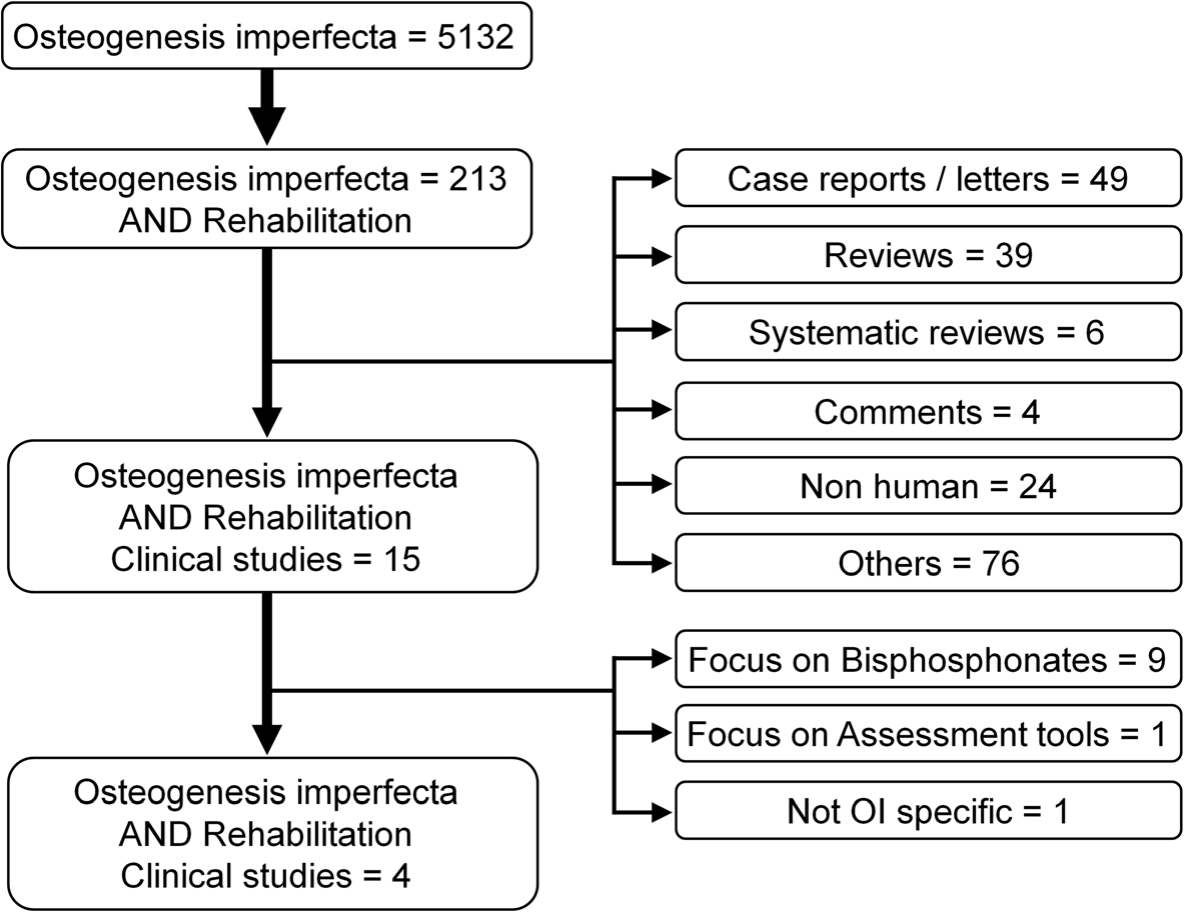
List of assessments used for children and adolescents with OI. ** are validated or specially developed for OI

Using the ICF as a framework helps to “determine if and how therapy may benefit the patient”. Assessment should be cover each domain of the ICF, clinical reasoning should underpin the individual-tailored treatment strategy, and where possible evidence-based. Various assessment tools, shown in Fig. 3, are used by different centers however only a few have been validated for OI. Most target other childhood onset conditions or healthy children and are not specific to OI. Many of these tools lack adaptations for short stature and impairments due to skeletal deformities. Test validated for OI are marked “**” in Fig. 3. In the future a standardized use of psychometrically valid and reliable instruments could facilitate research in this area.

The caregivers should be taught how to handle a child with OI from birth through to adulthood. Fear of fracturing resulting in inactivity and deconditioning remains a major issue which has to be addressed not only with the child but also with the caregivers. Some individuals with OI report that “overprotection” is a major problem, limiting their participation. Overprotection and the subsequent lack of muscle use leads to a reduced stimulus for bone formation and can create a “vicious cycle” of “fracture – pain – fear of movement – immobility- deconditioning – reduced skeletal stability – fracture” and needs to be targeted with an individually tailored therapeutic and in some cases psychological approach [14, 15].

Another important issue for individuals with OI is weight gain. Persons with reduced mobility risk becoming obese especially during puberty. Due to the occurrence of obesity even in those following a reduced caloric intake, a metabolic component to OI is frequently questioned but not yet proven. Currently it is recommended to monitor weight gain closely, try to avoid obesity and if necessary consult with a nutritionist because of the negative effects on mobility and self-care [16].

## Statement 1

The overall treatment goal for children with OI is to maximize mobility, function, activities and participation.

## Statement 2

A fear of fracturing is present in individuals with OI, their families and the health professionals treating them. This fear can be a limiting factor for reaching their full potential.

## Musculoskeletal

### Summary of the literature

Muscle and bones are connected anatomically and functionally. The collagen alteration related to OI affects the whole musculoskeletal system representing a constant challenge for children with OI. During training, the effect of muscles on the bone positively influences the areal bone mineral density (aBMD) [17]. Bone deformities, fractures, reduced muscle strength and length, alongside diminished BMD may affect body growth, motor development, level of independence and social participation. The detailed pathophysiology of the muscle weakness has yet to be elucidated but it is believed to be intrinsic to the impaired collagen (Veilleux et al., [18]).

There are differences in muscular mass and strength among children with OI, depending on OI type, age and functional level. A training program should therefore ideally take these variations into account and focus on the individual’s needs [7, 19–21].

Children with mild OI (type I) present with generalized hypermobility that decreases over time [7]. Only few have deformities of the long bones [22]. According to Pouliot-Laforte, children with OI type I have decreased muscle force and power, although they are as active as their healthy counterparts [23]. A proprioceptive deficit was revealed that could explain decreased postural control in this group. Caudill et al. showed weakness especially in the ankle plantar flexors that correlates with physical functioning like walking [24].

Van Brussel et al. found a 12-week individual and supervised training program in children with OI types I and IV to be safe and effective. Significant improvements in aerobic capacity by 18%, muscle strength by 12% and in level of fatigue were observed. These improvements diminished when children stopped performing exercises [11].

Approximately 70% of children and adolescents with moderate to severe OI also present with joint hypermobility [22]. They have mal-aligned lower and upper extremities and despite the hyperlax ligaments they also have a decreased total joint ROM due to deformities [7]. Muscle strength is decreased, and even more so in severely affected children. [14, 18].

## Standard of care

Alterations of the musculoskeletal system related to OI should be monitored regularly clinically or when appropriate radiologically. These include: coxa vara, genu valgum, leg length discrepancy, bowing, joint movement restrictions, joint hypermobility, muscular weakness, pain, disproportion, posture, fatigue, progressive deformities, scoliosis, etc.

When a supervised physiotherapy program is indicated, optional goals are to: promote motor development, improve/maintain aerobic capacity and muscle strength, reduce fatigue, relieve muscle-related pain, rehabilitate after fractures, and regain postural control [18, 25]. As comparison with healthy peers is not possible, interventions focus on improving disorder related physical functioning, performance and capacity. Also it is important to respect the variable clinical presentations of individuals with the different OI types. Weight-bearing activities, isometric exercises, muscular strengthening and functional activities are options in a rehabilitation program that should be based on physical assessment, knowledge of OI and clinical reasoning.

The major elements from the ICF domain ‘body structure’ related to muscle training are the improvement of muscle coordination and aerobic endurance. These elements are necessary to improve smoothness of movements and to give the individual a feeling of safety to prevent fractures [26]. Joint stability can be promoted through muscular strengthening, endurance and control training and proprioception activities. Active or active-assisted flexibility exercises may prevent or minimize restriction of joint movements and irreversible joint deformities. Gentle passive range of motion is not contraindicated but requires caution and an experienced professional. Orthoses can be prescribed to optimize mechanical musculoskeletal alignment.

An environment which stimulates the motor development of the child and which is also safe should be organized by parents, school and therapists. This can increase physical activity and improve function and level of independence.

## Expert experience

If there is an indication for a rehabilitation program, it must be based on the individual needs of the child/caregivers and a musculoskeletal and functional assessment. Furthermore the rehabilitation program should be evaluated by outcome measures. Physical fitness is decreased in children with OI, possibly due to inactivity or the decreased muscle strength inherent to the disease.

## Statement 3

Optimal muscle function can contribute to improve motor development, mobility and functional independence, as well as participation in society.

## Statement 4

After a fracture, active range of joint motion, muscular strength and function of the affected limb should always be re-evaluated. Early start of rehabilitation after a fracture is important to evaluate the functional impact of the fracture, intervening if necessary and avoiding immobility.

## Spine

### Summary of the literature

Deformities of the vertebral column develop during the first and second decade with a rapid progression during the pubertal age and may include scoliosis, lordosis and kyphosis. The reported incidence of spinal deformities varies between 40 and 85% depending on the severity of the disease [27]. A significant association between the onset of “supported sitting” and the degree of Cobb angle has been reported by Engelbert in children not treated with bisphosphonates. The mean age at occurrence of scoliosis was 7.0/6.8/9.0 years of age for OI Type I/III/IV [28]. Despite the positive effect of bisphosphonates on reshaping of compressed vertebrae during growth in children with OI [29, 30] the effect on the development of scoliosis remains controversial. [31, 32]. Anissipour showed a correlation between OI type and scoliosis with an incidence of nearly 100% in severely affected children. In moderately affected children scoliosis worsens during puberty [27]. In a small case series surgical procedures were shown to be effective in reducing the Cobb angle, if combined with previous halo traction [33]. A spinal fusion of only a few vertebrae is not sufficient to yield long term improvement [34].

## Standard of care

To date there is no empirical evidence that physiotherapy interventions can influence development of spinal deformities. However physiotherapy to strengthen trunk muscles and increase lung capacity may improve positioning and walking abilities of the patients and reduce chronic back pain.

Bracing in the treatment of spinal deformities in OI is controversial. There is no evidence in the literature that bracing leads to stabilization or improvement of scoliosis in OI. Bracing is often used for a few months following spine surgery to avoid displacement of the instrumentation. In mild OI bracing is prescribed in a similar fashion as with patients with adolescent idiopathic scoliosis, but no specific technique targeting the fragility of the vertebrae is known [10].

## Expert experience

The expert group agreed that no physiotherapy intervention has been shown to effectively treat vertebral deformities in OI. Different approaches such as postural education, positioning, or the Schroth method have been used to increase spine-pelvic alignment but have not yet shown distinct results.

## Statement 5

Strengthening of the trunk muscles and extremities may be used to decrease back pain, improve breathing capacity and trunk stability for sitting.

## Statement 6

Soft spinal braces have been used post-surgery to stabilize the trunk however with no evidence showing its efficacy in OI. Bracing in individuals with OI with spinal deformities is not yet recommended.

## Self-care and role of upper extremity

Self-care is defined as the essential tasks of taking care of one’s body such as eating, dressing, grooming, bathing, and management of oral and toilet hygiene and for purposes of this project include transfers (the ability to move oneself onto a bed, toilet, etc.).

## Summary of the literature

Individuals with severe OI have limitations in living independently due to upper extremity issues and so may require assistance with self-care tasks. Whereas individuals with OI type IV receiving a long-term multidisciplinary treatment approach usually had excellent function in self-care and transfer skills. They may be slower than their healthy peers to reach full independence, but gain independence through childhood [35]. Marr et al. describes the special problem of children with type V OI who struggle with restricted elbow range of movement due to calcification of the interosseous membrane and dislocation/subluxation of the radial head [36]. Several authors propose the provision of aids and adaptations as well as compensatory strategies in order to overcome the limited range of motion and/or muscle strength and so help the child with moderate to severe OI achieve independence [36].

Engelbert concluded that treatment should primarily focus on improving functional capability and adopting compensatory strategies, more than merely improving range of motion and muscle strength [37]. Montpetit suggests more advances in the clinical management of upper extremity issues are needed to mitigate the dependence resulting directly from deformities of the upper extremity [35].

## Standard of care

Children with OI should be encouraged to participate in self-care at the age appropriate time. Barriers to participation include environmental restrictions, short or bowed upper limbs with weakness and reduced range of motion. Children with mild to moderate OI are normally independent for self-care tasks.

Independent sitting and reaching are important pre-requisites to acquiring self-care skills. Compensatory strategies, home modifications and assistive devices can compensate for deceased elbow flexion or pronation/supination, and weak, bowed arms.

Non-ambulatory and children of short stature must learn how to move safely and independently to bed, toilet and tub/shower. Increased assistance with self-care and/or use of compensatory strategies will be required at times of fracture and immobilization.

## Expert experiences

The training of self-care has to be adjusted to the therapeutic aim agreed upon with the child and family. Depending on the tasks muscle training and the use of assistive devices should be combined. For dressing*,* a supine position on a large surface can ease the task and cotton material could be used to reduce friction. Modifications of clothes (large openings, elastic waist etc.) might be helpful. For dressing, bathing and toileting (perineal hygiene) assistive devices (dressing sticks, long handled shoe horn, bath sponge, toilet paper holder, etc.) can be useful for children with limited range of motion in shoulder and elbow joint. Children with OI are for the most part independent for feeding. Adequate seating should be available to position the child at an appropriate height surface. Non-slip mats and lightweight or contoured cutlery can aid those with weak or developing grasp. Some young children have issues with chewing and managing food textures. Speech and language therapists can provide support and advice as required.

The ability to transfer independently to chair, bed, toilet and bath is an essential aspect of self-care independence. Transfers require adequate muscle strength in the arms and legs. Additionally contractures may limit the use of devices and should be avoided as much as possible*.* Portable steps, commodes, benches, grab bars and transfer boards can resolve architectural barriers for bathing and toileting. It is important to ensure there is adequate space for the movements required and the use of devices. Intensive training with these adaptations is necessary to ensure the child is safe as well as independent.

## Statement 7

Upper extremity issues in children with OI may limit participation in daily self-care activities.

## Statement 8

Appropriate assistive devices, compensatory strategies and architectural adaptations can overcome the limited upper extremity range of motion and weak muscle strength and thus promote independence in self-care.

## Infant & Development

### Summary of the literature

The first years of life are critical for overall child development including motor, cognition, sensory processing and emotional regulation. According to Engelbert the severity of OI has a large influence on the age and sequence in the development of motor milestones [37]. Graff et al. concluded that even children with mild OI (type I) are smaller from the beginning than non-OI-children [16]. However motor development might be delayed depending on the severity of the disease. Those with a mild phenotype can reach normal motor milestones comparable to non-OI children [38] [22]. Infants with moderate to severe OI follow an individual developmental pathway, typically achieving developmental milestones later than non-affected infants with a discrepancy between static and dynamic milestones. Daly and associates studied the relation between motor milestones and the prognosis for walking. They found that independent sitting at the age of 10 months was a predictor of walking as the main means of mobility in 76% [39].

## Standard of care

The goal of therapy is to assist each child with OI to reach their maximum in developmental milestones and achieve level of independence while trying to prevent fractures and deformities. The therapist’s role is to: educate the caregiver in safe strategies for careful positioning and handling of infants to reduce the risk of fracture, how to handle a child in case of acute fracture, to prevent the secondary effects from immobilization due to fracture and to optimize musculoskeletal alignment for development. Therapists and caregivers strive for a child with OI to achieve as much functional independent mobility as possible. Any activity or mobility within proper precautions related to the diagnosis of OI will aid muscle and bone strengthening.

Physiotherapists and occupational therapists assess the infant with OI for: delays in motor development skills delays compared to other age equivalent infants, muscle weakness, or any signs of fracture or pain (crying, posturing or guarding of a limb). Motor delays may first be noticed in an infant with OI if there is a lack of physiological flexion, decreased active head, trunk and limb movements.

Therapists use standardized tools to assess body structure and function impairments, activity and participation limitations and personal and contextual factors that may contribute to the infant’s development.

## Expert experience

Facilitating development in infants with OI starts with typical interactions such as holding, feeding and caring as with any infant. Development can be affected by fractures, secondary effects of immobilization, fear to handle or move an infant with OI due to musculoskeletal, pulmonary, gastrointestinal issues and/or the caregiver’s tendency to overprotect the infant. Therapists should adhere to the pediatrician’s recommendations for positioning infants on their backs to sleep and tummy to play. Therapists may recommend alternate positioning options than supine when the infant is awake to prevent skull deformities, to encourage the development of antigravity active movements of the head, trunk and limbs as well as for exploration and mobility within his/her environment. Due to bone fragility, especially the rib cage, careful monitoring of the infant’s tolerance for these alternate positions is recommended. With the proper environment and equipment, many infants and children with OI can develop and grow to function well in most areas of daily life.

There is as yet no evidence as to what is the best care for infants with OI in the first year of life. However experts from specialized centers suggest similar approaches as described below:

Careful handling of infants with OI during routine activities such as bathing, diapering, breast/bottle feeding and dressing includes: using wide hand support, slow and gentle movements, avoid pulling, twisting of arms or legs or picking up the infant around the ribs. Handling and cuddling with caregivers should be encouraged. When carried the infant should be held close to the parent in a variety of positions, such as parent chest to infant chest. However, despite the most careful of handling, children with more severe OI will likely continue to fracture during infancy.

Appropriate baby equipment such as car seats, strollers and high chairs should have firm back support and a recline option. To avoid jarring or jerking movements which may increase risk of fracture, “baby bouncers”, “jumperoos”, swings, and baby walkers are not recommended.

Therapists assess the resting posture of infants with OI in various positions such as supine, prone, side lying. Infants may demonstrate a head rotation preference, alongside limbs that are externally rotated, abducted and in a flexed posture. Positioning may be done with towel rolls to maintain optimum spinal and extremity alignment and may prevent muscle contractures. Carefully monitor the infant’s tolerance in prone which should be avoided in the event of fractures. Extra care is needed for those infants with upper extremity deformities.

Sitting can be progressed from reclined, supported and unsupported positioning as the infant is developing head and trunk control. Even when an infant with OI can maintain sitting without falling, therapists and caregivers should note that protective extension responses may put the infant at risk for fracture and may be inefficient if bowing is present in the upper extremities. Sitting upright can be initiated when the infant has adequate head and trunk control. Head control may develop later than in age equivalent peers in non-OI infants. Infants with severe OI with delayed head and trunk control and/or with the presence of vertebral fractures may require inclined supported seating longer until the infant is able to maintain and/or achieve the sitting position independently.

When the infant with OI is trying to weight bear on their feet or starts to pull to stand, the therapist assesses the need for support of lower extremities. It is recommended to consult with an orthopedist regarding use of a standing frame, indication for rodding if lower limb bowing present, and need for orthotics or bracing of the lower extremities.

## Statement 9

Early physical rehabilitation of infants with OI includes assessment, therapy and caregiver education. Therapists educate caregivers on optimal and safe positioning and handling that facilitates nurturing and development while minimizing risk of fractures and deformities.

## Statement 10

Despite the most careful of handling, infants and children with OI will continue to fracture during infancy. Therapists and caregivers should use wide hand support, slow and gentle movements and to avoid twisting the limbs.

## Statement 11

Alternating positions (supine, prone, side lying) can minimize skull and limb deformities. It is important to initiate upright sitting only once the infant has adequate head and trunk control.

## Statement 12

Some infants with OI will follow a typical developmental course while others may follow an individual path, developing their own strategies for movement.

## Mobility

Functional mobility refers to floor mobility (moving in supine or snaking, rolling, bottom scooting), wheeled mobility (tricycle or bicycle, manual or power wheelchair), ambulation with or without ambulation aids and transfer or transition skills (the mobility needed to move from wheelchair or floor or standing to other surfaces and heights).

## Summary of the literature

Independent mobility is a critical requirement for living autonomously. Almost all children with mild OI are able to walk by about 2 years of age [38]. Engelbert and colleagues found that children with moderate to severe OI had less chance of ultimately walking compared to those with type I (mild) [40]. Possible causes include might be chronic pain or decreased ankle plantar flexor strength and can lead to significant limitations in sport and physical function [24]. Montpetit found that individuals with OI Type IV, receiving a long-term treatment approach usually achieved community ambulation and excellent transfer skills. In contrast none of the individuals with severe OI achieved independent ambulation [35]. In severely affected children providing appropriate wheelchairs and other mobility aids is essential for achieving independent mobility [36].

To improve mobility of ambulatory children, Caudill suggest a physiotherapy program of progressive strengthening including stair climbing, walking uphill, Theraband exercises, elliptical training, aquatic exercises, stationary cycling and Biodex training. The involvement in extracurricular recreational activities may also be beneficial [24]. Gerber et al. target hip extensors and abductors as well as spinal musculature strengthening in conjunction with pool therapy and bracing to ensure continued upright and ambulatory activity [26].

When physical training is part of an intensive rehabilitation approach with inpatient stays and home training, a significant increase of motor function and walking distance was seen in children with mild to severe OI as described above [12].

## Standard of care

The goal of physiotherapy and occupational therapy is to maximize the child’s potential for functional mobility thus facilitating participation in age appropriate activities. It is important to recognize the need for multiple mobility options depending on the environment (e.g. school versus home or indoor versus outdoor) and current status (e.g. fracture, surgery, fatigue). It is important to provide the appropriate mobility aids as indicated for the severity of the condition and type of mobility. For young children aids for floor mobility can be scooter boards, prone boards or mini-floor-wheelchairs. In terms of wheeled mobility the options include balance bicycles, tricycles and manual wheelchairs and power mobility devices. For children able to walk, there are ambulation aids, such as front or rear wheeled walkers. Additionally all kinds of walking canes (axilla or forearm crutches, quadrapod, tripod or single point canes) can be helpful. Walkers may have seats and/or forearm platforms to disperse upper extremity weight bearing in case of deformities or after fractures or surgery. Judicious use of lower limb orthoses may assist in promoting upright mobility and may be required during weight bearing after surgery.

When children demonstrate potential for independent ambulation, the rehabilitation intervention should start with household or indoor ambulation, then progress to outdoor ambulation. The use of ambulation aids initially can promote endurance and confidence and then be reduced when sufficient strength and fitness is present. Factors like head size, weight, height, rate of fractures and scoliosis and ambulation should be taken into consideration.

## Expert experiences

Most children with mild OI are able to walk medium distances in the community independently. Children with hypermobile, painful, or deformed feet with deformities may benefit from shoe orthotics, supramalleolar orthoses or ankle foot orthoses to provide sufficient support. Shoe lifts may be indicated to correct leg length discrepancies. The minimal amount of lower limb orthoses should be used to maximize muscle strength as much as possible.

Participation in activities involving high impact or contact sports should be assessed by carefully considering the specific activity and the individual. The high risk of fracture, falling and physical collision during the activity must be taken into account before providing a recommendation.

Children with moderate OI walk medium distances with or without ambulation devices and may benefit from a lightweight manual wheelchair during periods of frequent fractures, surgeries, or for safety during longer, unpredictable distances. They may choose to use power mobility in certain circumstances to manage uneven terrain, long distances or to allow participation in age appropriate activities (college, travel, etc). Therapists should be cautious about recommending power mobility for children with mild to moderate forms of OI who have excellent ambulation skills in order to prevent loss of muscle mass, aerobic capacity, and endurance.

Children with severe OI use wheeled mobility as their main method of moving, but may ambulate very short distances unless the musculoskeletal deformities are not correctable or the bone quality is unable to support their own body weight. However walking even very short distances also referred to as therapeutic ambulation is important for facilitating transfers from wheelchair to toilet, bed, car etc.

Therapeutic ambulation should be aimed for early in life and can maintain into adulthood making independent living easier. Some children use a wheelchair in the community (school, job, leisure) but being able to stand and take a few steps makes access to tub, toilet, and stairs at an entry possible. For those unable to walk, independent wheelchair propulsion (manual or electric one) is essential for independence, improving function and participation in social activities. All wheelchairs should fit the child appropriately. Seat depth and width should match the child’s femur length and hip width avoiding external rotation and abduction of hips. Children with severe OI can benefit from wheeled mobility as early as 18 months. When a wheelchair is provided at this young age it is necessary to continue working on the goal of ambulation (e.g. practice standing, do muscle strengthening and most importantly stretching of hip flexors which contract with extended sitting and become a major obstacle to ambulation).

## Statement 13

Most children with mild to moderate OI are able to walk independently with or without ambulation aids, however decreased muscle strength, fatigue and/or pain may limit endurance and/or involvement in sports.

## Statement 14

Children with OI should have access to a range of mobility aids to promote participation and independence. Orthotics can be considered to maximize mobility, optimize muscle function and minimize symptoms of pain and fatigue.

## Statement 15

The use of wheelchairs should be adjusted to meet the child’s participation needs and should not replace standing and walking activities. Wheelchairs should be chosen carefully to match the size of the child.

## Therapy after surgeries

### Summary of the literature

Shapiro states the goal of orthopedic surgery in OI is to help the bone grow straight, reduce fracture rate and in the event of fracture to prevent bone displacement [41]. Telescopic or elongating rods were initially developed by Bailey and Dubow [42], but have since undergone development by many surgeons [43–45].

Improved ambulation and functional ability are well known benefits of intramedullary rodding of the lower limb [6]. More recently improved function following humeral and forearm rodding have been documented [46]. Evidence of physiotherapy and occupational therapy management of this particular client group is extremely scant in the literature, with only two articles briefly outlining post-operative rehabilitation [6, 35].

## Standard of care

Surgical procedures are part of the multimodal approach to improve the situation of the child and the family. Surgery has to be incorporated alongside other treatments such as bracing in young children to prevent contractures and to achieve a more neutral position in orthoses (e.g foot position in case of contractures of the gastrocnemius), bisphosphonates, physiotherapy and occupational therapy and has to be adapted to the needs and possibilities of the family. Occupational therapy and physiotherapy with children who are having surgery can be divided into 3 episodes of care – pre-surgery, immediately post-surgery and ongoing rehabilitation. Additionally it is important to differentiate whether the child is undergoing rodding for the first time with correction of severe deformities or if a re-rodding due to growth or dislocation of rods.

Communication with patient and family, surgeon and multidisciplinary team, is essential in planning surgery and rehabilitation, understanding and setting goals and in adhering to the post-operative protocol. Pre-operatively baseline function, ROM, muscle strength and length, pain and QoL should be measured using standardized and validated outcome measures.

During the first few days/weeks after surgery the patient and their family must learn the use of compensatory strategies and assistive devices to manage any reduced mobility, promote self-care and reduce the need for assistance. Following surgery parents/caregivers should learn to help their child move safely, whilst in cast or while non-weight bearing. For ambulant children, early weight bearing should be encouraged using a walking aid as advised by the surgeon.

The rehabilitation process should focus on ROM (initially active assisted, then active), muscle strengthening, improvement of balance and proprioception, gait and functional re-education. Following cast removal, within the post-operative clinic or during physiotherapy review, any progress in rehabilitation is re- evaluated in line with patient/family goals and surgeon’s protocol.

Individuals with OI are at risk of developing hypertrophic scars. This can often be managed with standard scar management techniques. In severe cases a referral to a plastic surgeon maybe required.

## Expert experiences

Pre- surgery it is important for therapists to discuss details such as; timing of surgery, type and position of any splint/cast, level of mobility permitted (pre and post-operatively), aids/equipment for seating, self-care and transportation. Pre-operative standardized assessments of motor and functional skills, including current level of mobility, transfers and independence with activities of daily living allows objective assessment of surgical outcome and can increase satisfaction with the procedures.

During the immediate post-surgery period, rehabilitation should focus on reducing negative consequences of the immobilization. Where possible active and active assisted ROM exercises are encouraged in joints above and below operated segments. If agreed by the surgeon, muscle strengthening activities of both the operated and contralateral limb can prevent muscle atrophy.

For those patients vulnerable to complications, additional attention has to be paid to respiratory function and breathing techniques should be provided. Prior to discharge patients and families need to learn safe methods of transfer, with equipment if required, to prevent pressure areas in the case of lower extremity surgery, if possible early weight bearing should be promoted using walking aids.

During ongoing rehabilitation regular standardized assessment of functional outcome allows any improvement or attainment of pre-operative goals to be highlighted and could be used to increase motivation for further training.

## Statement 16

Well-coordinated, multidisciplinary management pre- and post-operatively, incorporating rehabilitation goals and equipment needs, ensures a quick return to functional activities and participation.

## Statement 17

Rehabilitation following lower extremity surgery should focus on ROM, muscle function, gait and functional re-education.

## Limitations

This paper combines the best available knowledge from experts in the field. The selection process was difficult and based on the criteria mentioned in the method section, but it still remained a bit random and depended also on their willingness to participate. Another limitation is that we can not guaranty not having overseen any performed research which was not published in the searched databases.

The most critical point is that this consensus only reflects the subjective opinions of the participants and that this is not a proper trial. Due to the lack of evidence nothing else was possible, but by bringing together people with different backgrounds and expertise we tried to limit this factor.

The Care4Brittle Bones foundation supported the process by providing their network to contact therapists in different countries and by organizing the administrative part of the consensus meeting. They provided a research grant to BM to support the literature research and the preparation of the consensus meeting and the publication. C4BB was not involved regarding the content of the statements. They will support us distributing this knowledge in the OI community in the future.

A further limitation is the restriction to children and adolescents and only on physical rehabilitation. This does not cover the whole area of rehabilitation which includes many other therapeutic strategies which could not be dealt with in this paper. Because the available evidence is even weaker in adults, this age group was neglected for this paper, knowing that physical activity remains an important part for people with OI throughout their whole life.

## Conclusion

This consensus paper combines the expert knowledge of 16 international experts in the field of rehabilitation in children and adolescents with Osteogenesis imperfecta. The review of the literature showed a severe lack of clinical trials. Therefore the experts developed 17 statements regarding physical training offering some guidelines to improve motor function in children with OI.

## Abbreviations

aBMD: Areal bone mineral density
HKAFO: Hip-knee-ankle-foot orthoses
ICF: International Classification of Functioning, Disability and Health
OI: Osteogenesis imperfecta
OIFE: Osteogenesis imperfecta Federation Europe
QoL: Quality of life
ROM: Range of motion
WHO: World Health Organization
